# Wrist pain: a systematic review of prevalence and risk factors– what is the role of occupation and activity?

**DOI:** 10.1186/s12891-019-2902-8

**Published:** 2019-11-14

**Authors:** R. Ferguson, N. D. Riley, A. Wijendra, N. Thurley, A. J. Carr, Dean BJF

**Affiliations:** 1 Nuffield Department of Orthopaedics, Rheumatology and Musculoskeletal Sciences (NDORMS), Botnar Research Centre, University of Oxford, OX3 7LD Oxford, England; 2 Nuffield Orthopaedic Centre, Oxford University Hospitals NHS Foundation Trust, Windmill Rd, Oxford, OX3 7LD UK; 3 Bodleian Health Care Libraries, Cairns Library, John Radcliffe Hospital, Headington, Oxford, OX3 9DU UK

**Keywords:** Wrist, Pain, Review, Epidemiology, Risk factors, Prevalence, Systematic review

## Abstract

**Objective:**

To evaluate the prevalence and risk factors of wrist pain.

**Methods:**

Systematic review. Data sources: The MEDLINE and EMBASE via OVID, CINAHL and SPORTDiscus via EBSCO databases were searched from database inception to 9th March 2018. Specific criteria were used to define inclusion and exclusion. Data was extracted independently by a pair of reviewers.

**Results:**

In total 32 cross sectional studies were identified for inclusion (1 with a longitudinal component). The median prevalence of wrist pain in the general population and non-manual workers within the short term (within last week) was 6 and 4.2% within the medium term (> 1 week and within a year). The median prevalence of wrist pain in physically demanding occupations and sports people was 10% within the short term and 24% within the medium term. Non-modifiable factors associated with wrist pain included increased age (1 study in adults and 3 studies in children/adolescents) and female sex (2 studies). Modifiable risk factors included high job physical strain (2 studies), high job psychological strain (1 study), abnormal physeal morphology in children/adolescents (2 studies), high frequency impact tool use (1 study) and effort reward imbalance (1 study).

**Conclusions:**

Wrist pain is highly prevalent in groups who partake in physically demanding activities from day to day such as manual labourers and sportspeople. It is less prevalent in the general population and non-manual workers, although there is a relative lack of research in the general population.

**Trial registration:**

The review protocol was registered with PROSPERO under the registration number CRD42018090834.

**Level of Evidence:**

1 (Prognostic study).

## Background

Musculoskeletal pain is a highly prevalent and costly health care problem globally [1]. Wrist pain accounts for an annual consultation prevalence rate of 58 in 10,000 patients in the UK [2], and is the fourth most common site of musculoskeletal pain in the upper limb after the shoulder, hand and elbow. While Walker-Bone et al. have demonstrated that non specific hand and wrist pain has a prevalence of around 10% in the general population [3]. Wrist pain is seen by a wide variety of clinicians in the United Kingdon including general practitioners, physiotherapists, occuptational therapists, sports doctors, orthopaedic surgeons, plastic surgeons and rheumatologists. Generally the management depends upon diagnosis reached, certain traumatic conditions are managed very differently to inflammatory conditions.

The factors associated with pain in the hand and the distal upper limb in general have been reviewed by other authors [4, 5], while other studies have reported on the prevalence of specific musculoskeletal problems in specific professions such as physicians and golfers [6, 7]. Other reviews have summarised the evidence relating to the whole upper limb [8], or have results which do not separate the wrist from the hand [9]. However we are unaware of any previous systematic review related to the epidemiological evidence relating to wrist pain as a specific entity. From a clinical perspective wrist pain and hand pain are very different entities, not only in terms of diagnosis but also in terms of management.

In this context our aim was to summarise the epidemiological evidence relating specifically to wrist pain. Specifically our aim was to perform a systematic review of the prevalence and risk factors of outcome of wrist pain in adults and children. All risk factors were sub grouped into the modifiable and non-modifiable categories.

## Methods

The systematic review was developed in accordance with the PRISMA statement, using the methods decribed in the Cochrane Handbook for Systematic Reviews of Interventions and modified as described here. The protocol was developed and peer reviewed locally before registration on the PROSPERO database (CRD42018090834).

### Data sources and searches

A comprehensive search strategy was created in collaboration with a research librarian (NT) and was designed to capture all relevant articles pertaining to observational studies relating to wrist pain (Additional file 1:). The full search strategy is detailed on the PROSPERO website. The search strategy was applied to the following bibliographic databases from database inception until 9th March 2018: MEDLINE and EMBASE via OVID, CINAHL and SPORTDiscus via EBSCO from database inception until 9th March 2018.

### Inclusion/exclusion criteria

The inclusion and exclusion criteria were defined prospectively during the protocol stage. Inclusion criteria included any cross sectional study or longitudinal study with a study population of any age and any setting with signs and/or symptoms of wrist pain reported within this group. There was no restriction on the type of setting for potential included papers. Included studies were required to report prevalence data, and had to be published in English or where an English translation was available. Exclusion criteria included: if the study population was defined on the basis of wrist pain (e.g.a solely asymptomatic and/or symptomatic group); if the study population was selected from a specific disease area (e.g. diabetes, rheumatoid arthritis, osteoarthritis); if patients with acute traumatic wrist pathology were deliberately included as new ‘incident’ cases (e.g. scaphoid fracture, distal radius fracture, scapholunate ligament rupture). Only studies which had asked participants specifically about wrist pain were included, studies which had amalgamated hand and wrist pain together in their questioning were excluded. Therefore wrist pain was defined as any pain attributed to the wrist by the patient or an observer/assessor, and pain attributed non-specifically to the wrist (for example to both the wrist and hand in a question or diagram) was not included within this definition. Studies in which the data had not been broken down to exclusively relate to wrist pain (for example by combining hand and wrist pain) were excluded. This underpinned the stated aim of the review which was to summarise information relating to wrist pain, not hand and wrist pain. Case reports and systematic reviews were excluded. A paediatric/adolescent population was defined as a population containing entirely members under the age of 18 years.

### Selection of studies

Duplicates were removed and relevant studies identified from the search were imported into Covidence for screening. Studies were independently screened by title and abstract by two authors (BD and RF). The references of all included studies and all relevant review articles on the topic were also reviewed to identify other potential studies for inclusion. This was followed by a full-text evaluation of the selected studies. Disagreement between the two reviewers was solved by consensus involving a third author (NR).

### Data extraction

Two reviewers (BD and RF) independently extracted data. Data was extracted using a custom data extraction sheet in Covidence (http://www.covidence.org). The data extracted included the author name, year of publication, journal, setting of study, type of study, population type and demographics, type of measurement used, prevalence of wrist pain, risk factors and predictive risk factors. Risk factors were defined as factors associated with wrist pain at one time point; while a predictive risk factor was defined as a factor which was assessed for predicting the development of wrist pain, meaning that a minimum of two time points would need to be studied. Risk factors were divided into the non-modifiable and modifiable groups. Any inconsistencies between the two reviewers’ forms were resolved by consensus discussion. A third review (NR) was available for any disagreement that could not be resolved by this initial discussion.

If data was not available from full-text articles or trial registrations, authors were contacted to provide this information. If authors were not contactable as regards additional data, then this aspect of the study was excluded from the data synthesis. If contactable authors did not respond to initial requests, they were sent two subsequent reminders over a minimum of 6 weeks. If there was still no response for the additional data, then this aspect of the study could not be included in the data synthesis.

### Outcomes

The prevalence and risk factors of wrist pain were of primary interest. The time frame over which incident wrist pain was reported was grouped as short term (current or up to and including past 7 days) and medium term (beyond 1 week and up to and including 1 year).

### Risk of bias assessment

Included studies were assessed for risk of bias by two independent raters (BD and NR) using a custom checklist based on that used by Lewis et al. [10]. It included six sections that assessed the study population, participant attrition, prognostic factor measurement, outcome measurement, confounding measurement, and statistical analysis. Each section had from 3 to 6 questions that were rated as high, low or unclear risk of bias (Additional File 4). Where appropriate, separate questions were used to evaluate studies which investigated risk factors and predictive risk factors. Any disagreements between ratings were resolved by discussion between the raters. A third party (NR) was available in any case where disagreements persisted after discussion. The checklist is attached a Additional file 2.

### Data analysis

Descriptive analysis was performed for all data to facilitate narrative interpretation and comparison across studies. We analysed the prevalence data by dividing it into six groups based upon the time period over which the wrist pain was assessed and the type of participant group (general population and non-manual workers, higher risk groups (physically demanding occupations and sportspeople) and children/adolescents. We excluded the data from studies which did not state the time period over which the prevalence of wrist pain was assessed over.

## Results

### Study selection

**Fig. 1:**
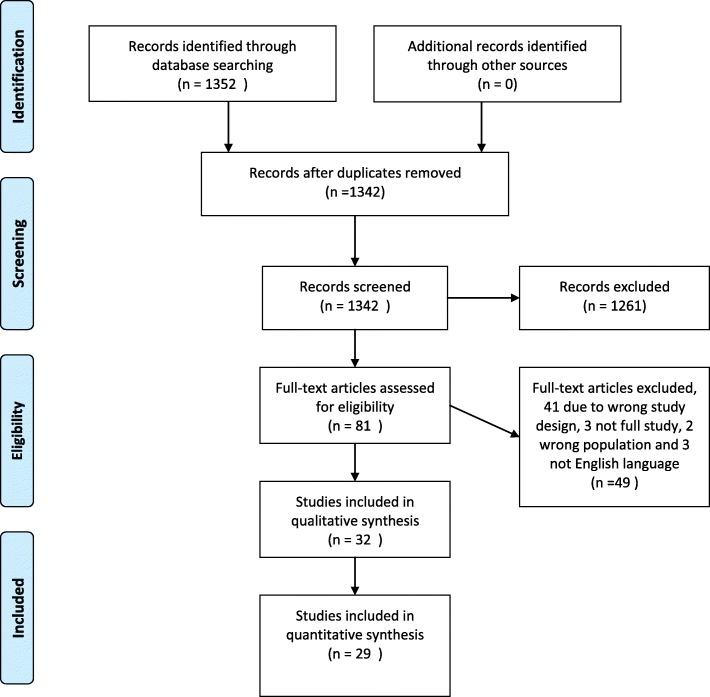
PRISMA flow diagram

A total of 1342 studies were identified by the search, after duplicates were removed. Following initial screening 82 studies remained for screening by full-text, 32 studies were then identified as eligible for inclusion (Fig. 1). The number of studies identified and excluded at each stage is detailed in Fig. 1.

### Study characteristics, results of individual studies and synthesis of results

Study characteristics of the included studies including the demographics, study design and wrist pain measurement method are provided in Table 1. The results are summarised in Tables 1 and 2. Table 1 details the prevalence of wrist pain. Table 2 details any extra information, as well as the associated factors and risk factors for wrist pain.

**Table 1:** Study characteristics

Author	Year	Journal	Setting	Population (number and type)	Age – mean (sd) unless stated	Sex	Type of study	Measurement of wrist pain/time period	Prevalence
Carnes et al [41]	2008	Family Practice	Community	2493 of general population	52 (range 18–102)	44% M 56% F	Cross sectional postal questionnaire	Chronic wrist pain, at least half the days in last year	Right wrist 5% Left wrist 4%
Celik et al [24]	2018	Dimensions of Critical Care Nursing	Hospital	111 critical care nurses	31.2 (5.8)	97 F 14 M	Cross sectional survey and interviews	Wrist pain using CMDQ (Cornell Musculoskeletal Disorder Questionnaire), over last year	40.5% right wrist 40.5% left wrist
Chang et al [26]	1995	Radiology	Opera school	326 students	14.2 (2.2)	137 M 189 F	Cross sectional questionnaire and radiographs	Questioned as to presence of wrist pain, not specified	52.5%
Das et al [17]	2014	International Journal of Occupational Medcine and Environmental Health	Workplace (office and brick fields)	220 brick field workers and 130 controls	35.5 (6.2) brick field 34.2 (6.7) controls	100% M	Cross sectional in 2 groups	Modified NMDQ (Nordic Musculoskeletal Disorder Questionnaire), not specified	85% (brick field workers) 3% (office workers)
Davatchi et al [15]	2008	The Journal of Rheumatology	Community	10,291 general population	30 (estimate from data provided)	51.1% M 48.9% F	Cross sectional	COPCORD Core Questionnaire (CCQ) wrist pain, in last 7 days	10%
Davatchi et al [42]	2015	Internal Journal of Rheumatic Diseases	Community	19,786 general population	30 (estimate from data provided)	0.9:1 M:F	Cross sectional	COPCORD Core Questionnaire (CCQ) wrist pain in last 7 days	10.4%
DiFiori et al [13]	2002	Clinical Journal of Sports Medicine	Gymnastics club	47 gymnasts	9.6 (range 5 to 16	26 M 21F	Cross sectional study	Wrist pain within last six months	57%
DiFiori et al [12]	2002	American Journal of Sports Medicine	Gymnastics club	59 gymnasts	9.3	31 M 28F	Cross sectional study	Wrist pain within last six months	56%
DiFiori et al [14]	1996	American Journal of Sports Medicine	Gymnastics club	54 gymnasts	11.8 (5.2)	21 M 33F	Cross sectional study	Wrist pain within last six months	73%
Gangopadhyay et al [18]	2007	Industrial Health	Workplace (brass metal workers and office)	50 brass metal workers 50 office	40.4 (6.4) brass 39.3 (4.1) office	100% M	Cross sectional in 2 groups	Wrist discomfort as modified NMDQ over last 12 months	62% brass metal workers 4% office
Harutunian et al [16]	2011	Med Oral Patol Oral Cir Bucal	Dental school	74 (54 students and 20 faculty)	28.9 (range 23–52)	47% M 53% F	Cross sectional	Questionnaire, wrist pain with last 6 months	27.1%
Hawkes et al [43]	2013	British Journal of Sports Medicine	Golf club	128 professional golfers	33.3 (6.3)	100% M	Cross sectional	Questionnaire, wrist problems currently	11.1%
Hou et al [44]	2006	Journal of Nursing Research	Hospitals	5169 nurses	30 approximately	100% F	Cross sectional	Modified NMDQ, wrist pain, over last year	10.5%
Inaba et al [45]	2011	Industrial Health	Workplace (goods sorting)	47 sorting cold goods 86 sorting dry goods	50.8	100% F	Cross sectional	Questionnaire, presence of wrist pain, over 4 month period	68% (cold goods) 41% (dry goods)
Jonasson et al [19]	2011	Knee Surg Sports Traumatol Arthrosc	Athletes and University staff	75 athletes 12 staff	Athletes 21.5 (range 10–40) Staff 28 (range 22–38)	100% M	Cross sectional in 2 groups	Questionnaire re presence of wrist pain, over last week and last year	9% athletes and 0% staff over last week 23% athletes and 9% staff over last year
Kihlberg et al [11]	2007	Int Arch Occup Environ Health	Workplace	680 workers who use power tools	Not stated	Not stated	Cross sectional	Questionnaire as regards current wrist pain	20%
Kirby et al [20]	2001	American Journal of Sports Medicine	Gymnasts and age matched controls	60 gymnasts 35 non gymnasts	11.8 (2.3)	100% F	Cross sectional in 2 groups	Questionnaire, time period not clear	33% gymnasts 2% non gymnasts
Kuwabara et al [21]	2011	World Journal of Gastroenterology	Hospital	190 endoscopists 120 non-endoscopists	41.4 (6.7) 40.1 (7.6)	261 M 49F	Cross sectional in 2 groups	Questionnaire, current presence of wrist pain	Right wrist – 2% endoscopists and 1% non endoscopists Left wrist – 7% endoscopists and 3% non endoscopists
MacDonald et al [46]	2014	Journal of Veterinary Cardiology	Veterinary echocardiographers	198 veterinary echocardiographers	40 approximately	50% M 50% F	Cross sectional	Questionnaire, wrist pain and time not stated	5.3%
McCue et al [47]	2004	Wilderness and Environmental Medicine	Fly-casting instructors	292 fly-casting instructors	Not stated		Cross sectional	Questionnaire, wrist pain for hours/weeks of the year or whole year	36.1%
Menzel et al [48]	2004	International Journal of Nursing Studies	Veteran’s hospital	113 nursing staff	42 (10.7)	13 M 100F	Cross sectional	Wrist pain using CMDQ (Cornell Musculoskeletal Disorder Questionnaire), over last week	Author contacted but data was not obtainable
O’Kane et al [49]	2011	Clinical Journal of Sports Medicine	Gyms in Seattle	96 competitive gymnasts	11 approximately	100% F	Cross sectional	Questionnaire regarding overuse injuries	9.2%
Punnett et al [22]	1985	Scand J Work Environ Health	Boston garment shop and hospital	162 garment workers 76 hospital employees	42 (12) 41 (12)	100% F	Cross sectional in 2 groups	Questionnaire on wrist pain, most days for one month over last year	16.8% garment workers 4.3% hospital employees
Purnell et al [50]	2010	Physical Therapy in Sport	Acrobatic gymnastics clubs	73 acrobatic gymnasts	13.8 (3.6)	4 M 69F	Cross sectional	Questionnaire on wrist problem, currently affects performance and has for 3 months or more	12.7%
Saxena et al [51]	2014	Asia-Pacific Journal of Public Health	Dentist practices	213 dentists	31.2 (7.34)	55.%M 44.6%F	Cross sectional	Questionnaire, wrist pain within last 12 months	17.8%
Smith et al [52]	2004	Australian Journal of Rural Health	Nursing school	260 nursing students	25.5 (8.7)	28 M 232F	Cross sectional	Questionnaire, wrist pain within last 12 months	12.7%
Sokas et al [23]	1989	American Journal of Industrial Medicine	Garment workers union	144 sewing machine operators 2822 controls	53.8 (range 31–68)	13 M 177F	Cross sectional in 2 groups	Questionnaire, wrist pain lasting at least a month	8.5% sewing machine operators 4.16% controls
Viljamaa et al [53]	2017	Medical Problems of Performing Artists	Finnish Musicians’ Union	920 musicians	45 (10)	179 M 182F	Cross sectional	Questionnaire, wrist pain in last 30 days and pain exceeding 30 days in last year	30% F 19%M (in last 30 days) 8%F 5%M (more than 30 days in last year)
Waikakul et al [54]	1999	The Pain Clinic	Rubber tree plantattions	2609 plantation workers	Not stated	1603 M 1006 F	Cross sectional	Interview, with chronic pain defined as more than 3 weeks with VAS 3 of more in a year	5.5% ulnar sided wrist pain
White [55]	2013	Animals	The Spay Neuter Industry Professionals (SNIP) in USA	219 veterinarians	41 (range 26 to 76)	22 M 196F (1 not stated)	Cross sectional	Wrist pain using modified CMDQ (Cornell Musculoskeletal Disorder Questionnaire), over last week	37.9% right wrist 17% left wrist
Woldendorp et al [56]	2018	Int Arch Occup Environ Health	Dutch professional orchestra	141 bassists (73 mono-instrument, 68 multiple)	34.7 (14.2) 35.3 (15.8)	86.3% M 13.7%F 91.2%M 8.8%F	Cross sectional	Wrist pain, within 3 months and > 3 months/VAS severity	19.1% right wrist always/often within 3 months 24.1% left wrist always/often within 3 months
Yu et al [25]	2013	Industrial Health	Chinese factories and companies	5339 employees	35 approximately	3632 M 1706F	Cross sectional	Wrist pain as defined as more than 24 h in last year	33.5%

**Table 2:** Risk factors

Author	Year	Extra information	Risk factors – non-modifiable	Risk factors - modifiable
Celik et al [24]	2018	Minor vs slight vs major impact on work with right wrist pain 48.9% vs 33.3% vs 17.8% and with left wrist pain 48.9% vs 33.3% vs17.8%		Nurses who often lifted/carried heavy materials felt significantly more pain in the wrist (37.8%; OR, 0.17; 95% CI, 0.05–0.49; *P* = .003)
Chang et al [26]	1995	No increased risk of wrist pain with increased ulnar variance		24.6% of the 171 painful wrists had abnormal growth plate morphology compared to 19 (10.5%) of the 181 asymptomatic wrists (*p* < 0.005 X2 test, RR – 2.3)
Das et al [17]	2014			Higher risk of wrist pain in brick field workers (85%) versus office workers (3%) (p < 0.001, X2 test)
Davatchi et al [15]	2008		Wrist pain more common in women 14.7% (CI 13.6–15.8) than in men 5.6 (4.9–6.3)	
DiFiori et al [13]	2002	Wrist pain was dorsal (56%), palmar (22%), radial (7%) and ulnar (7%). Multivariate logistic regression analysis revealed this age range to be significantly associated with wrist pain, independent of training intensity, age of initiation of training, years of training, gender, height, and weight (p = 0.03). The 1-year changes in height and training intensity were not associated with wrist pain (*p* = 0.15 and *p* = 0.2, respectively).	Wrist pain was significantly more common in the older and taller groups. Pain free group mean age was 9.6 versus 11.3 in the painful group, *p* = 0.01. Pain free group mean height was 131.6 versus 139.6 in the painful group, *p* = 0.04). Of those between 10 and 14 years of age at 1 year, 73% had wrist pain at the study onset and at 1 year, compared with 29% of those who were either less than 10 or more than 14 years of age. (*p* = 0.004).	
DiFiori et al [12]	2002	By using multivariate regression analysis, we found that training hours per week (*P* = 0.03) and wrist pain (*P* = 0.02) were independently associated with radiograph findings of grade 2 or 3. Sixty-seven percent of the gymnasts (22 of the 33) with wrist pain had findings of grade 2 or 3, compared with 31% (8 of 26) of those without wrist pain (*P* = 0.008).	Age was the only independent risk factor for wrist pain after adjusting for confounders using multivariate regression modelling. Ulnar variance was not associated with wrist pain or radiographic injury of the distal radial physis	Wrist pain prevalence was associated with the radiographic grading of the distal radial physis (*P* = 0.007).
DiFiori et al [14]	1996	Wrist pain was dorsal (61.5%), palmar (7.7%), radial (6.2%) and ulnar (12.3%).	Two non-modifiable factors were independently associated with wrist pain (age > 10 years, *p* = 0.018;; age > 14 years, *p* = 0.016).ars (P = 0.016),	One modifiable factor was independently associated with wrist pain (training intensity, *p* = 0.036).
Gangopadhyay et al [18]	2007			Higher rate of wrist pain in brass metal workers (62%) versus office workers (4%) (*p* < 0.001 Chi squared test)
Harutunian et al [19]	2011	Of 27.1% with wrist pain, 20.3% were classified as mild, 4.1% moderate and 2.7% severe	Wrist pain was more common in females (*p* < 0.05)	Wrist pain was more common in those specialising in oral surgery (p < 0.05).
Hawkes et al [43]	2013	The majority of injuries (67%) occurred in the leading wrist at the most common location, the ulnar side of the wrist (35%). 87% of all ulnar-sided and 100% of radial-sided problems were in the leading wrist.	N/A	
Hou et al [44]	2006	Total of 3.4% had a limitation of movement due to the wrist pain reported. Wrist pain increased risk of sick leave OR 2.96 (95% CI 2.06–4.20) adjusted OR 2.36 (95% CI 1.60–3.42).	N/A	
Jonasson et al [19]	2011	Note inconsistency between figures in text and tables.	. Significant associations noted between presence of wrist pain versus thoracic spine pain *p* = 0.0188 OR 17.60 (95% CI 1.73–178.76) and wrists versus hips *p* = 0.0437 OR 12.00 (95% CI 1.63–88.29). Also significant associations noted over last year of symptoms related to elbows versus wrists *p* = 0.0026 OR 16.50 (95% CI2.51–108.64) and wrists versus thoracic spine *p* = 0.0508 OR 6.56 (95% CI1.17–36.84).	Higher rate of wrist pain in athletes versus staff
Kihlberget al [11]	2007		Higher risk of wrist pain with age (OR 1.4 (95% CI 1.1–1.7)).	Higher risk of wrist pain with high frequency impact tool use (OR 1.5 (95% CI 1.0–2.3)).
Kirby et al [20]	2001			Higher rate of wrist pain in gymnasts (33%) versus non gymnasts (2%)
Kuwabara et al [21]	2011			Higher rate of wrist pain in endoscopists versus non endoscopists
MacDonald et al [46]	2014	41.9% incidence of wrist pain reported whilst carrying out echocardiograms	N/A	
McCue et al [47]	2004	Of those reporting wrist pain, 51% indicated it lasted for hours, 29% indicated it lasted for days, 6% indicated it lasted for weeks, and 4% indicated it lasted all year.		Chi-square tests revealed significant differences in wrist pain prevalence between the overhead and the sidearm styles (32% vs 49%, *P* = 0.01), between the overhead and the elliptical styles (32% vs 58%, *P* = 0.03), between the sidearm and the multiple styles (49% vs 20%, *P* = 0.05), and between the elliptical and the multiple styles (59% vs 20%, *P* = 0.03).
Menzel et al [48]	2004	The frequency of wrist discomfort was predicted by number of highest risk tasks per hour and number of patients ≥212 pounds .	N/A	
Punnett et al [22]	1985			Wrist pain more common in garment workers versus hospital employees RR 3.9 *p* = 0.005 (95% CI 1.4–10.9). Specific types of garment workers were extremely likely to experience wrist pain (Finishers, RR 8.5)
Saxena et al [51]	2014		There was no significant association between wrist pain and age.	There was no significant association between wrist pain and use of assistant, use of fitness regime and breaks.
Sokas et al [23]	1989			Wrist pain was significantly more common in sewing machine operators than controls (*p* = 0.00001).
Woldendorp et al [56]	2018			There was no statistically significant difference in the rate of wrist pain within the last 3 months when comparing mono-instrumentalists with multi-instrumentalists (*p* = 0.831 right wrist, *p* = 0.845 left wrist)
Yu et al [25]	2013			Wrist pain was more common in men and women with high job strain (psychological demands) (men OR 1.4 (95%CI 1.02–1.91) and women OR 2.20 (95%CI 1.31–3.69)) and high job strain (physical demands) (men OR 1.37 (95%CI 1.05–1.80) and women OR 1.56 (95%CI 1.02–2.40)). Wrist pain was more common in men and women with a effort reward imbalance (ERI) (men OR 1.29 (95% CI 1.02–1.23) and women OR 1.56 (95% CI 1.00–2.42). Wrist pain was more common in women in relation to job control OR 1.37 (95% CI 1.07–1.75). Wrist pain was more common in men related to effort OR 1.25 (95% CI 1.05–1.47).

### Study characteristics

Of the 32 included studies, all of which were cross sectional studies (1 with a longitudinal component); seven of these studies compared two distinct populations while the remainder analysed only one population. Seven studies related to solely children and adolescents, while the remaining 25 studies related solely to adult populations. The studies by Davatchi et al. and Fiori et al. do have overlap in terms of the population group studied, although they have described different results relating to these populations. The method of assessing the prevalence of wrist pain was highly variable. The CMDQ (Cornell Musculoskeletal Disorder Questionnaire) was used by three studies, the COPCORD Core Questionnaire (CCQ) by two studies and a version of the NMDQ (Nordic Musculoskeletal Disorder Questionnaire) by three studies. The other methods for assessing wrist pain are summarised in Table 1. The time frame over which wrist pain was assessesed was also highly variable, varying from ‘current’ to pain within the last year as is detailed in Table 1.

### Results – prevalence

**Fig. 2:**
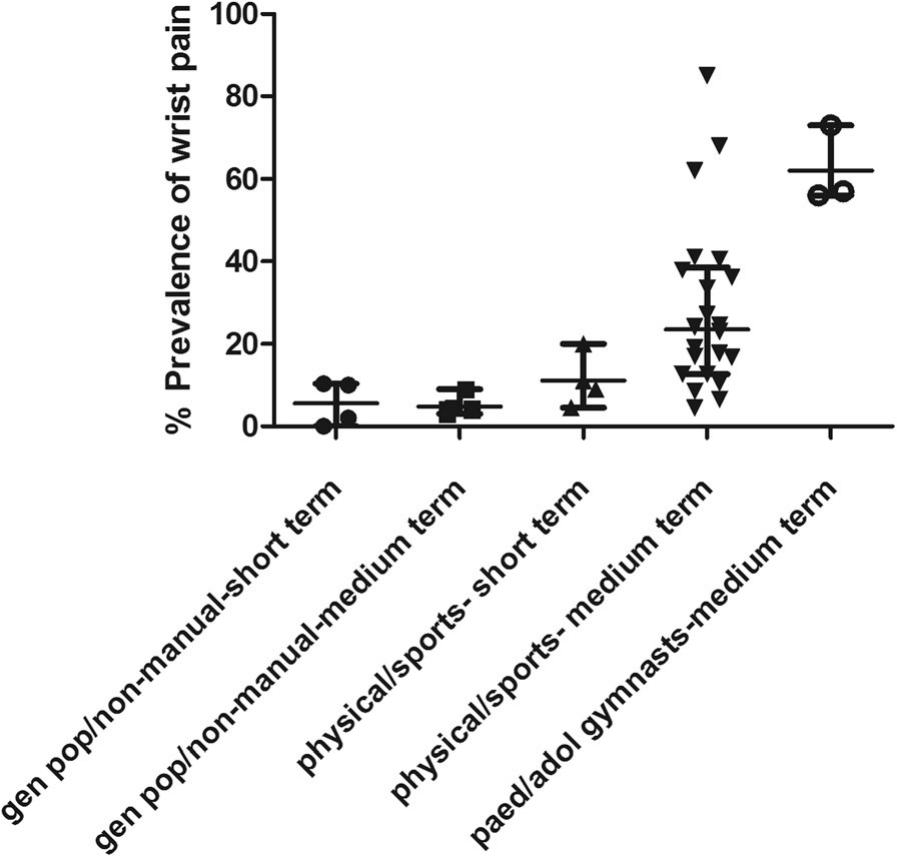
Scatter plot depicting the prevalences of wrist pain in different groups. The line and bars represent the median and interquartile range for each group

These results are detailed in Table 1 and shown in Fig. 2. The median prevalence of wrist pain in all populations combined within the short term (within last week) was 10% (IQR 3.3 to 15.6) and 19.1% (IQR 8.5 to 40.5) within the medium term (> 1 week and within a year).

The median prevalence of wrist pain in the general population and non-manual workers within the short term (within last week) was 6% (IQR 0.5 to 10.3) and 4.2% (IQR 43.75 to 5.6) within the medium term (> 1 week and within a year). The median prevalence of wrist pain in physically demanding occupations and in sportspeople was 10% (IQR 5.6 to 17.8) within the short term and 24% (IQR 12.7 to 38.6) within the medium term. The median prevalence of wrist pain in the medium term in female child/adolescent gymnasts was 57% (IQR 56 to 73). Figure 2 represent a scatter plot of the prevalence of wrist pain in the different groups, we wish to make it clear this is not a form of meta-analysis. The prevalence of wrist pain was much lower in the non-gymnastic general paediatric population as reported by Kirby et al., however no time frame was reported for the wrist pain so this was not included within the scatter plot [11].

### Results – risk factors

These results are detailed in Table 2, while Fig. 2 shows the prevalence of wrist pain in the different groups.

### Non-modifiable risk factors

The non-modifiable factors associated with wrist pain included increased age (1 study in adults [12] and 2 studies in children/adolescents [13–15]), and female sex [16, 17]. Kihlberg et al. demonstrated an odds ratio of 1.4 (95% CI 1.1–1.7) with increased age [12], while the studies by Di Fiori et al. did not provided an odds or risk ratio, Davatchi et al. showed that the frequency of wrist pain was 14.7% (CI 13.6–15.8) in women, higher than in men 5.6% (CI 4.9–6.3), however no odds or risk ratio was provided [16]. Harutunian et al. found a higher prevalence of wrist pain in women but provided no further data relating to the strength of this association [17].

### Modifiable risk actors

The impact of occupation was investigated by several studies. The prevalence of wrist pain was in higher in brick field (85%) vs officer workers (3%) [18], brass metal (62%) vs officer workers (4%) [19], athletes vs university staff [20], gymnasts (33%) versus non gymnasts (2%) [11], endoscopists versus non endoscopists [21], garment workers vs hospital employees (RR 3.9 (95% CI 1.4–10.9) [22], and sewing machine operators versus controls [23].

The modifiable factors associated with wrist pain included high job physical strain ( [24, 25], 2 studies), high job psychological strain [25], abnormal physeal morphology in children/adolescents (2 studies [13, 26]), high frequency impact tool use [12] and effort reward umbalance [25]. Yu et al. demonstrated that wrist pain was more common in men and women with high job strain (psychological demands) (men OR 1.4 (95%CI 1.02–1.91) and women OR 2.20 (95%CI 1.31–3.69)) and high job strain (physical demands) (men OR 1.37 (95%CI 1.05–1.80) and women OR 1.56 (95%CI 1.02–2.40)); wrist pain was also more common in men and women with a effort reward imbalance (ERI) (men OR 1.29 (95% CI 1.02–1.23) and women OR 1.56 (95% CI 1.00–2.42, 25). Celik et al. nurses who often lifted/carried heavy materials felt significantly more pain in the wrist (37.8%; OR, 0.17; 95% CI, 0.05–0.49). Chang et al. found that 24.6% of the 171 painful wrists had abnormal growth plate morphology compared to 19 (10.5%) of the 181 asymptomatic wrists (RR 2.3, 26). While Kihlberg et al. found a higher risk of wrist pain with high frequency impact tool use (OR 1.5 (95% CI 1.0–2.3)) [12].

### Predictive risk factors

Only one study assessed predictive risk factors and this was observed at a follow up time of five years, demonstrating that in workers who use power tools a higher rate of wrist pain at 5 years associated with high frequency impact tool use (RR 1.6 (95%CI 0.8*–*3.4)) and number of years in occupation (RR 1.5 (95% CI 0.9*–*2.5)) [12].

### Risk of bias within studies and across studies

**Fig. 3:**
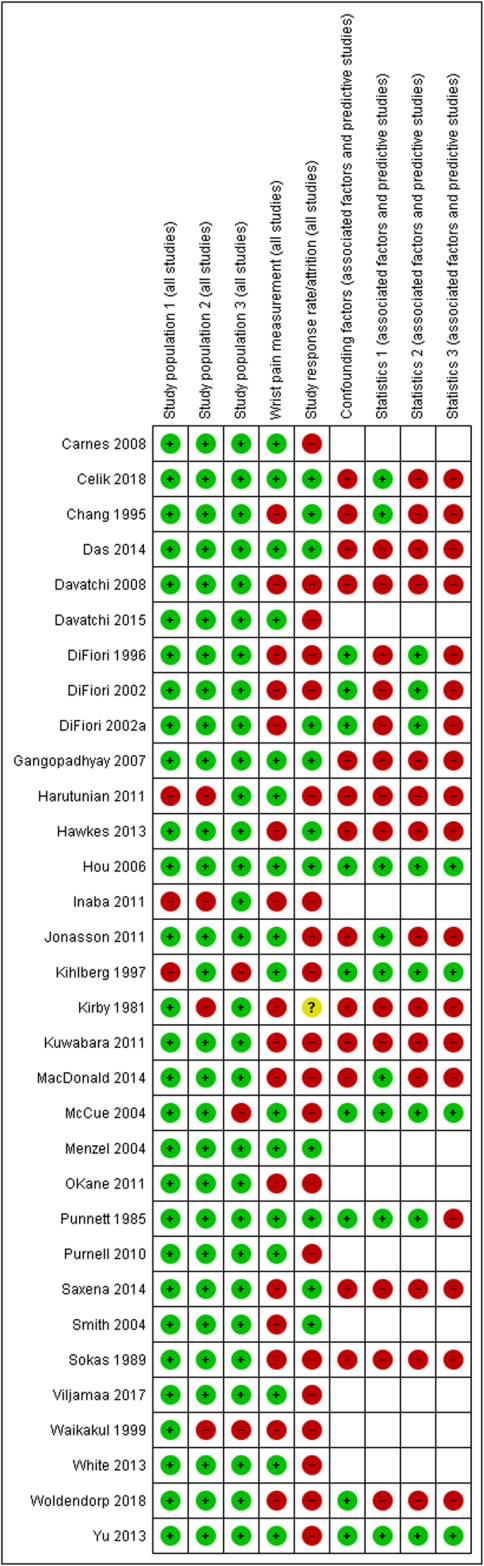
Risk of bias summary. Review authors’ judgements about each risk of bias item for each included study

The risk of bias summary is shown in Fig. 3 and the risk of bias graph in the Additional file 3. The risk of bias was generally low for the study population domains (description of sampling, inclusion/exclusion criteria and reporting of basic participant characteristics). In terms of response rate and wrist pain measurement the risk of bias was higher on average, with a majority of studies judged to be at high risk of bias in these domains. Again the results were mixed in the confounding and statistics domains for studies which investigated associated factors. A significant proportion of studies investigating the associated factors did not report odds ratios or risk ratios with their 95% confidence intervals, as well as those adjusted for confounding. Only one study assessed the risk factors of wrist pain and was scored against the relevant domains for prognostic studies.

## Discussion

The key finding of this systematic review is that wrist pain is highly prevalent in groups who partake in physically demanding activities from day to day such as manual labourers and sportspeople. It is less prevalent in the general population and non-manual workers, although there is a relative lack of research in this area. It is also pertinent that there is a lack of epidemiological research investigating the relationship between structural abnormalities and pain in adults.

There is a significant body of evidence which demonstrates that modifiable risk factors such as occupation, workplace demands and sporting activity are associated with wrist pain [11, 12, 18–25]. This is consistent with the evidence relating to other sites of chronic musculoskeletal pain such as the shoulder and spine [27–30]. While Da Costa et al. have shown that heavy physical work, smoking, high body mass index, high psychosocial work demands increase the risk of work related musculoskeletal disorders [31]. In this review only one study assessed predictive risk factors, demonstrating that workers who use power tools have a higher rate of wrist pain at 5 years, this is associated with high frequency impact tool use and the number of years in occupation. This points to the importance of the holistic approach in assessing and managing patients with wrist pain, as it may be useful to detect specific modifiable risk factors which can be incorporated into any potential treatment plan.

Although wrist pain is not as common as back, shoulder, hip and knee pain, it nonetheless represents a significant proportion of the overall musculoskeletal burden [2]. While there is epidemiological evidence to demonstrate a relationship between structural abnormalities in hip and shoulder pain for example [32, 33], this review has found no epidemiological evidence that demonstrates a clear relationship between structural change and wrist pain in adults. This is problematic as in the absence of the epidemiological evidence to demonstrate that specific structural abnormalities are associated with pain and dysfunction, there should be significant uncertainty regarding the treatment of any form of chronic wrist pain with a surgical intervention in order to address structure. The studies by DiFiori et al. in young gymasts are the only ones within this review which have shown that a structural abnormality, abnormal physeal morphology, is associated with wrist pain [13, 26].

The prevalence of radiographic wrist osteoarthritis varies within the scientific literature. Studies by Kellgren and Van Saase both demonstrated a prevalence of radiographic wrist osteoarthritis of around 5 to 10% in men women, [34, 35]. A lower prevalence was reported in the Framingham study of less than 2% [36]. These differences may well relate to different radiographic thresholds used for determining the presence of radiographic osteoarthritis. While other structural abnormalities around the wrist have been shown to be highly prevalent in asymptomatic patients such as those relating to the TFCC [37], extensor carpi ulnaris tendon [38] and ganglia [39, 40]. In this context it is unsurprising that the results of surgery can be unpredictable when treating structural abnormalities which are highly prevalent in the asymptomatic general population. Generally degenerative structural change is far more common with increasing age and it is salient in this review that only one study demonstrated that age was an associated factor for wrist pain [12]. This means that highly prevalent structural abnormalities are unlikely to be a significant explanatory factor for wrist pain in general adult populations.

### Limitations

The main limitations of this systematic review relate to the included studies’ limitations. There are significant methodological flaws present within the included studies. These include the use of unvalidated methods of assessing wrist pain, the low response rates and the lack of adjustment for confounding factors in some studies. Another significant limitation is the number of studies (*n* = 41) which had to be excluded due to study design (Fig. 1), this was largely down to the way in which wrist pain had not been specifically investigated. As previously stated our specific aim was to assess wrist pain as a distinct entity and this underlies the exclusion of studies which did not separate hand and wrist pain.

## Conclusions

Overall there is a lack of high quality research investigating the epidemiology of wrist pain. The existing evidence demonstrates that wrist pain is highly prevalent in groups who partake in physically demanding activities from day to day such as manual labourers and sportspeople, while it is less prevalent in the general population and non-manual workers.. There is also a lack of research investigating the relationship between structural abnormalities and pain in adults which would be a sensible target for future research.

## Supplementary information

**Additional file 1.** Full search histories.

**Additional file 2.** PRISMA checklist.

**Additional file 3.** Risk of bias graph. Review authors’ judgements about each risk of bias item presented as percentages across all included studies.

**Additional file 4.** Details of the risk of bias domains against which studies were deemed at ‘high’, ‘low’ or ‘unclear’ risk of bias.

## Data Availability

All data underlying the results are available as part of the article and no additional source data are required.

## Abbreviations

CCQ: COPCORD Core Questionnaire
CMDQ: Cornell Musculoskeletal Disorder Questionnaire
IQR: Interquartile range
NMDQ: Nordic Musculoskeletal Disorder Questionnaire
TFCC: Triangular fibrocartilage complex
UK: United Kingdom
