# Supplementary material for: Wrist pain: a systematic review of prevalence and risk factors– what is the role of occupation and activity?
Source: BMC Musculoskelet Disord. 2019 Nov 14;20:542. doi: 10.1186/s12891-019-2902-8 (PMC6857228; doi:10.1186/s12891-019-2902-8)
Supplement: Supplementary file 1 — Additional file 1. Full search histories. [file 12891_2019_2902_MOESM1_ESM.docx]

Database: Ovid MEDLINE(R) Epub Ahead of Print, In-Process & Other Non-Indexed

Citations, Ovid MEDLINE(R) Daily and Ovid MEDLINE(R) <1946 to Present>

Search Strategy: 09/03/18

--------------------------------------------------------------------------------

1 Wrist/ (8083)

2 wrist*.ti,ab. (32896)

3 Wrist Injuries/ (5878)

4 WRIST JOINT/ (9070)

5 radioulna*.ti,ab. (2042)

6 Triangular Fibrocartilage/ (327)

7 triangular fibrocartilage*.ti,ab. (898)

8 tfcc.ti,ab. (467)

9 ulnocarpal.ti,ab. (262)

10 druj.ti,ab. (426)

11 pisotriquetral.ti,ab. (96)

12 extensor carpi ulnaris.ti,ab. (513)

13 flexor carpi ulnaris.ti,ab. (660)

14 De Quervain Disease/ (184)

15 de quervain*.ti,ab. (711)

16 first dorsal compartment*.ti,ab. (95)

17 intersection syndrome.ti,ab. (51)

18 radiocarpal.ti,ab. (1313)

19 (ulnar* adj3 abutment*).ti,ab. (18)

20 (ulnar* adj3 impaction*).ti,ab. (163)

21 scaphotrapezoidtrapezoidal*.ti,ab. (0)

22 scaphotrapezial*.ti,ab. (87)

23 kienboch*.ti,ab. (12)

24 preiser*.ti,ab. (68)

25 (avascular necro* adj3 scaphoid* adj3 idiopathic).ti,ab. (19)

26 exp CARPAL BONES/ (8309)

27 carpal*.ti,ab. (15007)

28 capitate.ti,ab. (993)

29 hamate.ti,ab. (792)

30 lunate.ti,ab. (1953)

31 pisiform.ti,ab. (477)

32 scaphoid*.ti,ab. (4294)

33 trapezium.ti,ab. (893)

34 trapezoid*.ti,ab. (3448)

35 triquetrum.ti,ab. (415)

36 1 or 2 or 3 or 4 or 5 or 6 or 7 or 8 or 9 or 10 or 11 or 12 or 13 or 14 or 15 or 16

or 17 or 18 or 19 or 20 or 21 or 22 or 23 or 24 or 25 or 26 or 27 or 28 or 29 or 30 or

31 or 32 or 33 or 34 or 35 (59064)

37 1 or 2 or 3 or 4 or 5 or 6 or 7 or 8 or 9 or 10 or 11 or 12 or 13 or 14 or 15 or 16

or 17 or 18 or 19 or 20 or 21 or 22 or 23 or 24 or 25 (43018)

38 exp PAIN/ (354456)

39 pain*.ti,ab. (592518)

40 38 or 39 (732512)

41 36 and 40 (9419)

42 37 and 40 (7404)

43 Epidemiologic studies/ (7620)

44 exp case control studies/ (900325)

45 exp cohort studies/ (1717262)

46 Case control.tw. (106211)

47 (cohort adj (study or studies)).tw. (150416)

48 Cohort analy$.tw. (6051)

49 (Follow up adj (study or studies)).tw. (44611)

50 (observational adj (study or studies)).tw. (78915)

51 Longitudinal.tw. (200460)

52 Retrospective.tw. (416938)

53 Cross sectional.tw. (270763)

54 Cross-sectional studies/ (258827)

55 43 or 44 or 45 or 46 or 47 or 48 or 49 or 50 or 51 or 52 or 53 or 54 (2527488)

56 41 and 55 (3278)

57 42 and 55 (2542)

58 incidence/ (226792)

59 incidence.ti,ab. (647349)

60 prevalence/ (248093)

61 prevalence.ti,ab. (520287)

62 exp risk/ (1048288)

63 risk*.ti,ab. (1833149)

64 factor*.ti,ab. (2874058)

65 epidemiolog*.ti,ab. (326859)

66 58 or 59 or 60 or 61 or 62 or 63 or 64 or 65 (5162881)

67 56 and 66 (975)

68 57 and 66 (761)

Database: Embase <1974 to 2018 March 08>

Search Strategy: 09/03/18

--------------------------------------------------------------------------------

1 *wrist/ (6861)

2 wrist/ (24590)

3 *wrist pain/ (175)

4 wrist pain/ (1242)

5 exp *wrist injury/ (6489)

6 wrist injury/ (4122)

7 wrist*.ti,ab. (43426)

8 *radioulnar joint/ (432)

9 radioulnar joint/ (1215)

10 radioulna*.ti,ab. (2250)

11 *triangular fibrocartilage/ (162)

12 triangular fibrocartilage/ (462)

13 triangular fibrocartilage*.ti,ab. (979)

14 tfcc.ti,ab. (518)

15 ulnocarpal.ti,ab. (315)

16 druj.ti,ab. (449)

17 pisotriquetral.ti,ab. (95)

18 extensor carpi ulnaris.ti,ab. (588)

19 flexor carpi ulnaris.ti,ab. (757)

20 *De Quervain tenosynovitis/ (166)

21 De Quervain tenosynovitis/ (385)

22 de quervain*.ti,ab. (794)

23 first dorsal compartment*.ti,ab. (98)

24 intersection syndrome.ti,ab. (58)

25 radiocarpal.ti,ab. (1524)

26 (ulnar* adj3 abutment*).ti,ab. (27)

27 (ulnar* adj3 impaction*).ti,ab. (173)

28 scaphotrapezoidtrapezoidal*.ti,ab. (0)

29 scaphotrapezial*.ti,ab. (98)

30 kienboch*.ti,ab. (4)

31 *Kienboeck disease/ (631)

32 Kienboeck disease/ (799)

33 Kienboeck*.ti,ab. (53)

34 preiser*.ti,ab. (78)

35 (avascular necro* adj3 scaphoid* adj3 idiopathic).ti,ab. (20)

36 1 or 5 or 7 or 8 or 10 or 11 or 13 or 14 or 15 or 16 or 17 or 18 or 19 or 20 or 22

or 23 or 24 or 25 or 26 or 27 or 28 or 29 or 30 or 31 or 33 or 34 or 35 (51888)

37 2 or 6 or 7 or 9 or 10 or 12 or 13 or 14 or 15 or 16 or 17 or 18 or 19 or 21 or 22

or 23 or 24 or 25 or 26 or 27 or 28 or 29 or 30 or 32 or 33 or 34 or 35 (53524)

38 exp *carpal bone/ (3986)

39 exp carpal bone/ (7855)

40 carpal*.ti,ab. (17805)

41 capitate.ti,ab. (1126)

42 hamate.ti,ab. (882)

43 lunate.ti,ab. (2187)

44 pisiform.ti,ab. (552)

45 scaphoid*.ti,ab. (4689)

46 trapezium.ti,ab. (989)

47 trapezoid*.ti,ab. (3986)

48 triquetrum.ti,ab. (482)

49 36 or 38 or 40 or 41 or 42 or 43 or 44 or 45 or 46 or 47 or 48 (69730)

50 37 or 39 or 40 or 41 or 42 or 43 or 44 or 45 or 46 or 47 or 48 (72583)

51 exp *pain/ (370443)

52 exp pain/ (1125018)

53 pain*.ti,ab. (848892)

54 51 or 53 (981571)

55 52 or 53 (1414854)

56 49 and 54 (18380)

57 50 and 55 (21981)

58 3 or 56 (18393)

59 4 or 57 (22311)

60 Clinical study/ (154286)

61 case control study/ (122548)

62 Family study/ (25461)

63 Longitudinal study/ (109036)

64 Retrospective study/ (617523)

65 Prospective study/ (427959)

66 Randomized controlled trials/ (140418)

67 65 not 66 (423617)

68 Cohort analysis/ (348466)

69 (Cohort adj (study or studies)).mp. (218474)

70 (Case control adj (study or studies)).tw. (110675)

71 (follow up adj (study or studies)).tw. (57244)

72 (observational adj (study or studies)).tw. (120808)

73 (epidemiologic* adj (study or studies)).tw. (95338)

74 (cross sectional adj (study or studies)).tw. (156272)

75 60 or 61 or 62 or 63 or 64 or 67 or 68 or 69 or 70 or 71 or 72 or 73 or 74

(2001893)

76 exp *incidence/ (18260)

77 exp incidence/ (366399)

78 exp *prevalence/ (50633)

79 exp prevalence/ (598910)

80 prevalence.ti,ab. (717645)

81 exp *risk/ (245282)

82 exp risk/ (2101339)

83 risk*.ti,ab. (2591447)

84 factor*.ti,ab. (3668664)

85 epidemiolog*.ti,ab. (413050)

86 76 or 78 or 80 or 81 or 83 or 84 or 85 (5880022)

87 77 or 79 or 80 or 82 or 83 or 84 or 85 (6557145)

88 58 and 75 and 86 (876)

89 59 and 75 and 87 (1189)

***************************
