# Supplementary figures and images for: Wrist pain: a systematic review of prevalence and risk factors– what is the role of occupation and activity?
Source: BMC Musculoskelet Disord. 2019 Nov 14;20:542. doi: 10.1186/s12891-019-2902-8 (PMC6857228; doi:10.1186/s12891-019-2902-8)

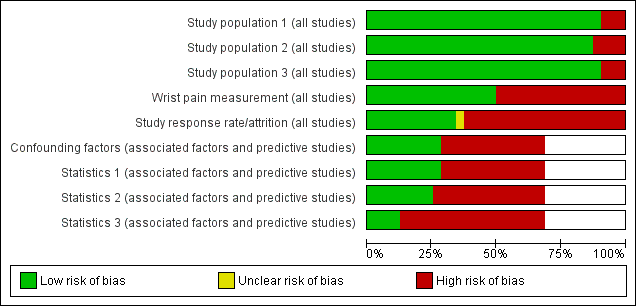

Supplement: Supplementary file 3 — Additional file 3. Risk of bias graph. Review authors’ judgements about each risk of bias item presented as percentages across all included studies. [file 12891_2019_2902_MOESM3_ESM.png]
