# Supplementary material for: Wrist pain: a systematic review of prevalence and risk factors– what is the role of occupation and activity?
Source: BMC Musculoskelet Disord. 2019 Nov 14;20:542. doi: 10.1186/s12891-019-2902-8 (PMC6857228; doi:10.1186/s12891-019-2902-8)
Supplement: Supplementary file 4 — Additional file 4. Details of the risk of bias domains against which studies were deemed at ‘high’, ‘low’ or ‘unclear’ risk of bias. [file 12891_2019_2902_MOESM4_ESM.docx]

Risk of bias tool – all criteria judged as high, low or unclear risk of bias for each domain

Study population 1 - Description of the sampling frame, recruitment, period and location of recruitment

Study population 2 - Inclusion/exclusion criteria clearly outlined?

Study population 3 - Important basic characteristics of the sample are reported

Study response rate/attrition - Complete data for at least 80% of the initial study sample size?

Study attrition 2 (predictive studies) - Reasons for loss to follow-up reported?

Study attrition 3 (predictive studies) - The basic characteristics of those who were lost to follow-up are reported?

Study attrition 4 (predictive studies) - There are no important differences between those who were lost to follow-up and those who completed the study

Wrist pain measurement - Clear definition of the outcome is provided. The outcome is measured using valid and reliable instruments.

Prognostic factor measurement 1 (predictive studies) - Clear definition or description of the predictive factors?

Prognostic factor measurement 2 (predictive studies) - Predictive factors measured by valid and reliable instruments?

Outcome measurement 1 (predictive studies) - Clear definition of the outcome is provided including duration of follow-up?

Outcome measurement 2 (predictive studies) - The outcome is measured using valid and reliable instruments?

Confounding factors (risk factors and predictive studies) - Has confounding been considered? Multivariate analysis used to adjust for potential confounding variables? Confounders are measured using valid and reliable methods?

Statistics 1 (risk factors and predictive studies) - Were measures of association estimated according to this model OR/RR (including 95% CI) and numbers in the analysis (totals) were presented?

Statistics 2 (risk factors and predictive studies) - Was there an explanation of how confounding factors were controlled for?

Statistics 3 (risk factors and predictive studies) - Are both unadjusted and adjusted (ie for confounding) results given if appropriate?

Statistics 4 (predictive studies) - Was the method of variable selection for regression modelling reported and justified? Was the number of variables included in the model appropriate and/or justified? Was the multivariate model prospectively validated?
